# Case Report: Treatment of Penile Intraepithelial Neoplasia in an Asian Male With Reconstructive Plastic Surgery

**DOI:** 10.3389/fsurg.2021.667417

**Published:** 2021-06-28

**Authors:** Kai Huang, Shuai Lu, Yehua Wang, Xiangan Tu

**Affiliations:** ^1^Department of Urology, College of Clinical Medicine, Northern Jiangsu People's Hospital, Yangzhou University, Yangzhou, China; ^2^Department of Urology and Andrology, The First Affiliated Hospital of Sun Yat-sen University, Guangzhou, China

**Keywords:** penile neoplasms, surgery, plastic, Asian, case report

## Abstract

Intraepithelial neoplasia is a special type of squamous cell carcinoma occurring in the skin epidermis. The incidence of penile intraepithelial neoplasia in Asian males is rare. We report the clinical characteristics and treatment process of a case of penile intraepithelial neoplasia in a Chinese man. We treated the disease of this patient by surgical excision of the penile lesion and scrotal flap plastic surgery. After surgery, the shape of the penis was satisfactory, and there was no adverse effect on erection. The pathological results confirmed the diagnosis of penile intraepithelial neoplasia. The pathological features showed that the lesion tissue was covered with squamous epithelium and that there was severe atypical hyperplasia of the lesion epithelium, disordered arrangement of polarity, and an intact basement membrane. The removal of the lesions of penile intraepithelial neoplasia through a wider surgical resection range, combined with the stretchability of a scrotal flap, can achieve a good healing effect of the surgical wound and reduce the possibility of recurrence of penile intraepithelial neoplasia.

## Introduction

First reported by Bowen (1), intraepithelial neoplasia is an *in situ* epithelial squamous cell carcinoma of the skin that generally does not infiltrate the dermis or surrounding tissues. Intraepithelial neoplasia most commonly presents in the proximal skin of the trunk or limbs and can also be located in the oral mucosa, conjunctival membrane, and nail bed (2). The etiology may be related to chemical factors (such as arsenic agents) and human papillomavirus (3). Few cases of penile intraepithelial neoplasia in Asian males have been reported. In November 2019, we treated one Chinese patient with penile intraepithelial neoplasia who underwent penile skin lesion excision and scrotal flap repair plastic surgery. The treatment effect was satisfactory. Now, we report the treatment of this patient as follows.

## Case Presentation

**Figure 1:**
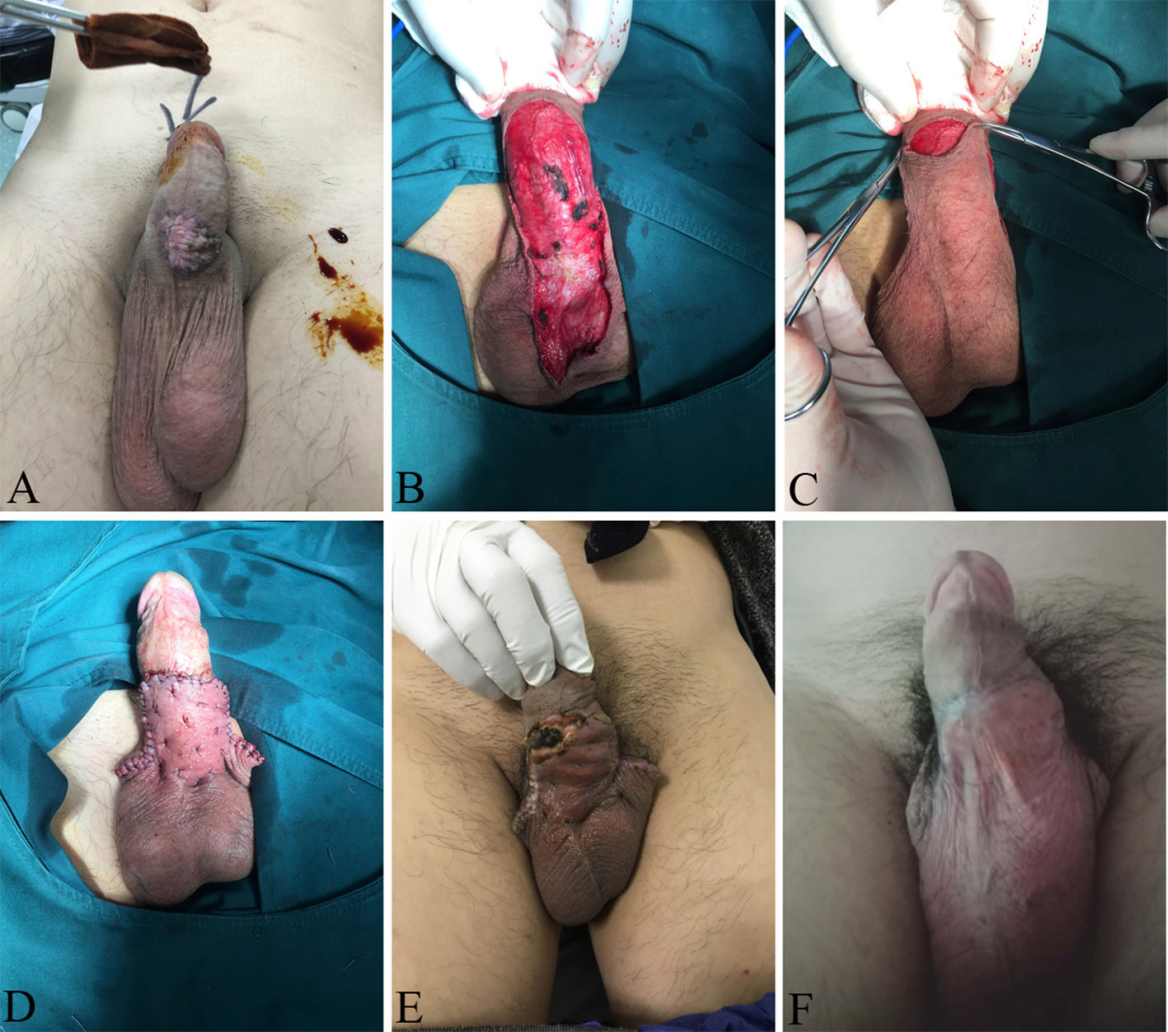
Intraepithelial neoplasia lesion located on the ventral side of the penis **(A)**. Complete resection of the lesion tissue on the surface of the penile deep fascia **(B)**. The penile surgical area was covered with a pedicled scrotal flap **(C)**. The shape of the penis after lesion resection and plastic surgery **(D)**. The surgical area of the penis of the patient is not completely healed half a month after the operation **(E)**. Penile morphology after complete wound healing **(F)**.

A 44-year-old Asian male patient gradually developed a ventral mass on the penis 7 years before presentation. Initially, the lesion was ~1 cm in diameter and occasionally accompanied by paresthesias and bleeding after friction. He had normal urination and did not visit the hospital at first. When an ulcer developed, he applied erythromycin ointment. Sometimes, the focal ulceration

healed, but the condition often recurred. The scope of the lesion gradually increased over the period of 7 years. Finally, he came to the dermatology department of our hospital and underwent penile lesion tissue biopsy. The pathological report showed that he had penile intraepithelial neoplasia. Considering the large range of lesions, he came to the urological ward for hospitalization and surgical treatment. The patient had no clear history of exposure to chemical properties such as arsenic. His HIV antibody test was negative. He did not have a history of smoking or alcoholism. The lesion was located on the ventral side of the penis and the total area was ~2.2 cm ×2.0 cm (Figure 1A). The surface of the penile lesion was pale red with a gray edge. It had an irregular shape and clear boundaries, and the lesion slightly protruded out of the surface of the skin. There was no obvious exudate on the ulceration, and there was no obvious redness or swelling of the surrounding skin.

After continuous epidural anesthesia, a skin incision line was marked at a 1.0 cm margin from the edge of the lesion of the penis of the patient. The scrotal flap range that needed to be isolated was also set before resectioning of the lesion. We cut the skin of the lesion vertically along the designed incision margin line, deep to the deep fascia layer, and completely resected the lesion tissue on the surface of the deep fascia (Figure 1B). After careful hemostasis by bipolar coagulation, we designed a 5.0 cm ×3.0 cm scrotal flap based on the resected wound area and dissociated it on the surface of the deep fascia. With a formed pedicled scrotal flap, we stretched it to the ventral side of the penis and covered the surgical area of the penile skin (Figure 1C). Intermittent suturing of the subcutaneous tissue reduced cavity formation and local exudate accumulation (Figure 1D). The wound area was dressed in gauze under pressure, and the penis was wrapped with a self-adhesive bandage under appropriate tension to promote wound healing. No drainage tube was placed in the operational area. The bandage and gauze were removed to expose the wound and keep it dry 3 days after surgery.

**Figure 2:**
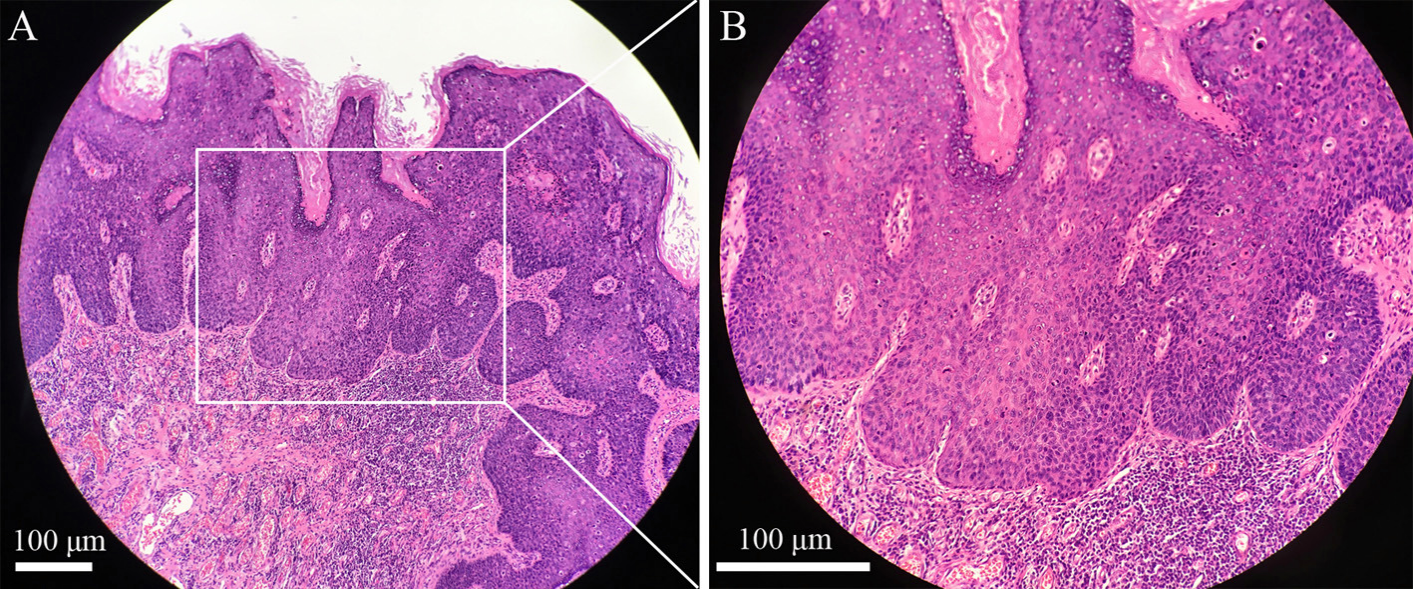
Pathological image of the penile intraepithelial neoplasia lesion with H&E staining, original amplification ×100, scale bar 100 μm **(A)**. Pathological image of the penile intraepithelial neoplasia lesion with H&E staining, original amplification ×200, scale bar 100 μm **(B)**.

After penile lesion excision, the wound was covered by scrotal flaps with blood supply. There was postoperative edema in the penile wound without infection. At follow-up observation half a month after the operation, the wound edge did not heal completely because of tension (Figure 1E). Through care with wound dressing changes, the wound area finally achieved healing (Figure 1F). Pathological images (Figures 2A,B) showed that the lesion tissue was covered with squamous epithelium and showed finger-like protrusion. The basal part of the lesion showed bulbous advancing growth. The epithelium in the lesion area had severe dysplasia, disordered arrangement of polarity, large nucleus, deep staining, and complete basement membrane. No definite infiltration of basal layer tissue was found. The lesion area showed infiltration of polymorphonuclear giant cells and lymphocytes. The pathological diagnosis was penile intraepithelial neoplasia with granulomatous inflammation. No diseased tissue was found at the incision margin. After 6 months of follow-up, the patient had no recurrence of penile intraepithelial neoplasia and had no obvious discomfort with penile erection.

## Discussion

The incidence of intraepithelial neoplasia is very low, and it is rare in Asian male penises. It usually occurs in people aged 60–70 years old. The lesions are mostly located in the head, neck, hands, trunk, buttocks, oral cavity, and nail bed. It is extremely rare in the penis. The lesions are characterized by one or several raised plaques with clear borders, squamous epithelioid changes, and uneven surfaces. Sometimes, it is easily misdiagnosed as eczema or psoriasis. Diagnosis is usually confirmed by skin biopsy. The cause of the disease is not clear at present. It is assumed to be related to the following pathogenic factors (4): arsenic poisoning, excessive ultraviolet exposure, and human papillomavirus infection. Sometimes, the lesions occurring in the penile skin need to be differentiated from invasive squamous cell carcinoma of the penis. In most cases, even small invasive tumors need active surgical treatment and expanded resection of the scope and depth of the lesions. This requires a sufficient depth for definitive diagnosis when taking lesion tissue for biopsy.

It is generally considered that intraepithelial neoplasia lesions can be surgically resected 5 mm around the lesion (5). Sufficient depth should be removed to prevent lesions in the case of invasive tumors. Considering the integrity of lesion excision and the extensibility of scrotal flaps, we designed an incision 1 cm away from the edge of the lesion in this patient with penile intraepithelial neoplasia. We resected the underlying tissue and sarcolemma of the lesion. The final results showed, that although the resection range was large, it was covered by scrotal flaps, and the surgical wound achieved good healing. The scrotal skin has great extensibility and little restriction on the extension when the penis is erect. It reduces the effect on penile appearance and function to the greatest extent after plastic surgery repair of the operational wound. This is an ideal surgical method for repairing the skin defect of penile wounds. Because the lesions are located in the epidermis and rarely invade the deep part, smaller intraepithelial neoplasia lesions are often treated with radiotherapy, cryotherapy (6), laser therapy, photodynamic therapy (7), or 5-fluorouracil ointment (8). However, the recurrence rate is higher after these treatments. For this patient, we thought that the choice of surgical treatment was more reasonable because of the large penile lesion area. A larger extent and depth of surgical resection can help to ensure the integrity of lesion resection and prevent recurrence of penile intraepithelial neoplasia. Some studies suggest that (9) the extent of surgical resection depends on the size of the tumor and the depth of invasion. Generally, the lesions not only rarely involve the subcutaneous and alveolar membrane but also are not accompanied by lymph node metastasis in the inguinal region.

For surgical resection of lesions, local pressure bandage or indwelling negative pressure drainage tube is used to reduce the accumulation of wound exudate. Considering the large range of lesions in this patient, it is easy to form local exudate accumulation and may lead to surgical wound infection. We used intermittent sutures of subcutaneous tissue to reduce the formation of the cavity and local exudate accumulation accompanied with a local appropriate pressure bandage. There was no drainage tube placed in the wound area. After the operation, the blood supply of the flap was good and there was edema on the penile wound without infection. About 6 months after the operation, the wound healed well.

After 6 months of follow-up, the Asian male patient had no recurrence of penile skin lesions. His penile erectile function was not significantly affected. The treatment of this patient with penile intraepithelial neoplasia shows that it can promote healing of the surgical wound by surgical excision of the lesion and plastic surgery by using a scrotal skin flap. It also provides useful surgical treatment planning for the surgical repair of penile skin defects, wound exposure, and other diseases in the case of trauma or surgery.

During the treatment of this patient, we also found that there were some deficiencies in the treatment effect. Although the scrotal flap was helpful for the healing of the surgical wound, it also caused hair attachment on the skin surface of the penis, which may arouse certain inconvenience during the sexual life of the patient. Due to the small number of such cases, this report only shares the treatment process and surgical experience of this patient. As for the treatment of penile intraepithelial neoplasia in the future, we need to accumulate more cases to help exploring and establishing standard diagnoses and treatment procedures that may contribute to achieving better treatment effects for patients.

## Data Availability Statement

The raw data supporting the conclusions of this article will be made available by the authors, without undue reservation.

## Ethics Statement

Written informed consent was obtained from the individual(s) for the publication of any potentially identifiable images or data included in this article.

## Author Contributions

KH: design of study, date collection, the surgeon who performed the operation, and manuscript writing. SL: the surgeon involved in the operation. YW: design of study and the surgeon involved in the operation. XT: design of study and manuscript revising. All authors contributed to the article and approved the submitted version.

## Conflict of Interest

The authors declare that the research was conducted in the absence of any commercial or financial relationships that could be construed as a potential conflict of interest.
